# The onset of cerebral infarction may be affected by differences in atmospheric pressure distribution patterns

**DOI:** 10.3389/fneur.2023.1230574

**Published:** 2023-07-31

**Authors:** Atsushi Fukunaga, Hideki Koyama, Takahisa Fuse, Asami Haraguchi

**Affiliations:** Department of Neurosurgery, Fussa Hospital, Tokyo, Japan

**Keywords:** cerebral infarction, embolism, thrombosis, atmospheric pressure distribution pattern, onset

## Abstract

**Background:**

Some papers have highlighted a possible causal relationship between the onset of ischemic stroke and weather conditions. This study aimed to elucidate the onset mechanism of cerebral infarction from a meteorological approach. We focused on the atmospheric pressure distribution patterns (APDPs).

**Methods:**

The subjects are 221 cases diagnosed as cardiogenic cerebral embolism (Group A) and 612 cases diagnosed as atherosclerotic cerebral thrombosis (Group B). We investigated the APDP on the date closest to the date and time of onset of cerebral infarction in each patient on the website and chose the most similar one from the reported 11 APDPs. Groups A and B were compared for clinical characteristics and the appearance rate of each APDP in each group.

**Results:**

The clinical characteristics of Groups A and B were consistent with some previously reported clinical characteristics of cerebral embolism and cerebral thrombosis except for smoking. The appearance rate of the other high-pressure type, which cannot be classified as either the anticyclone belt type or the migratory anticyclone type, in Group B was statistically significantly higher than that in Group A, and the appearance rate of the anticyclone belt type in Group A was statistically significantly higher than that in Group B (*p* < 0.05, Fisher's exact probability method, respectively).

**Conclusions:**

Cerebral embolism and cerebral thrombosis exhibited significant differences in APDPs on the day of onset. Dehydration particularly in the other high-pressure type or in the anticyclone belt type should be prevented. Further investigation should focus on the other meteorological factors.

## 1. Introduction

Cerebrovascular disease is Japan's fourth leading cause of death, following malignant neoplasm, heart disease, and senility. The statistics from the Ministry of Health, Labor and Welfare in Japan^1^ in 2020 show that there were 56,864 deaths (45.1 mortality rate per 100,000 population) due to cerebral infarction, which was higher than 31,997 deaths due to intracerebral hemorrhage and 11,416 deaths due to subarachnoid hemorrhage, although recombinant tissue plasminogen activator (rt-PA) therapy and/or thrombectomy is gaining more attention for acute ischemic stroke. The reduction of the mortality rate of cerebral infarction remains among the critical issues for a super-aging society like Japan.

Virchow's triad is a concept originally based on venous thrombus formation and has been known as a mechanism of thrombus formation. In arterial thrombus formation, the weighting of each factor is considered to be different from venous thrombus formation, but dehydration is a common causal factor of thrombosis and embolism. Dehydration causes an increase in protein concentration in the blood, resulting in blood stasis. On the other hand, human beings live under the influence of various weather changes, and can become ill under certain weather conditions, such as being prone to heatstroke under high temperature and humidity. In addition, in an anticyclone belt type, one of the atmospheric pressure distribution patterns (APDPs) in Japan, fine weather and dry air continue for several days or more, so there is a high possibility that we will easily become dehydrated if we neglect to replenish water. Therefore, we consider that there may be a link between weather changes and the Virchow's triad including the concept of dehydration. Some recent papers have highlighted a possible causal relationship between the onset of ischemic stroke and weather conditions (1, 2). Hence, this study aimed to elucidate the onset mechanism of cerebral infarction from a meteorological approach and to consider preventive methods against cerebral infarction. We focused on the APDPs peculiar to Japan in meteorology.

## 2. Methods

### 2.1. Subjects

Cerebral infarction is roughly categorized into cerebral embolism and cerebral thrombosis, and since each onset mechanism varies, cerebral infarction patients were split into cerebral embolism and cerebral thrombosis and compared with each other. The subjects are 221 cases diagnosed as cardiogenic cerebral embolism^2^ (Group A) and 612 cases diagnosed as atherosclerotic cerebral thrombosis^3^ including lacuna cerebral infarction^4^ (Group B) among the patients admitted to Fussa Hospital^5^, Tokyo, Japan, between October 2008 and November 2020. Our hospital is a designated secondary emergency hospital^6^, and accepts stroke patients in the Nishitama district of Tokyo, altitude 123 m, with a population of approximately 150,000. The time of onset was confirmed by hearing from the patient or the first discoverer such as the patient's family, and specifying the hour and minute. The distinction between embolism and thrombosis was made comprehensively according to the TOAST classification (3) and the A-S-C-O/ASCOD phenotypic classification (4, 5), based on the presence of heart disease such as atrial fibrillation (AF), electrocardiogram and echocardiographic findings, the number, location, and size of cerebral infarct lesions on head MRI images, and atherosclerotic stenotic findings in intra-/or extracranial arteries on head and neck MRA images. Some rare cerebral infarction cases such as those resulting from arterial dissection or unknown embolism origin, and cases on the unknown onset date and time were excluded. The number of cases excluded was 121.

### 2.2. Study design

**Figure 1:**
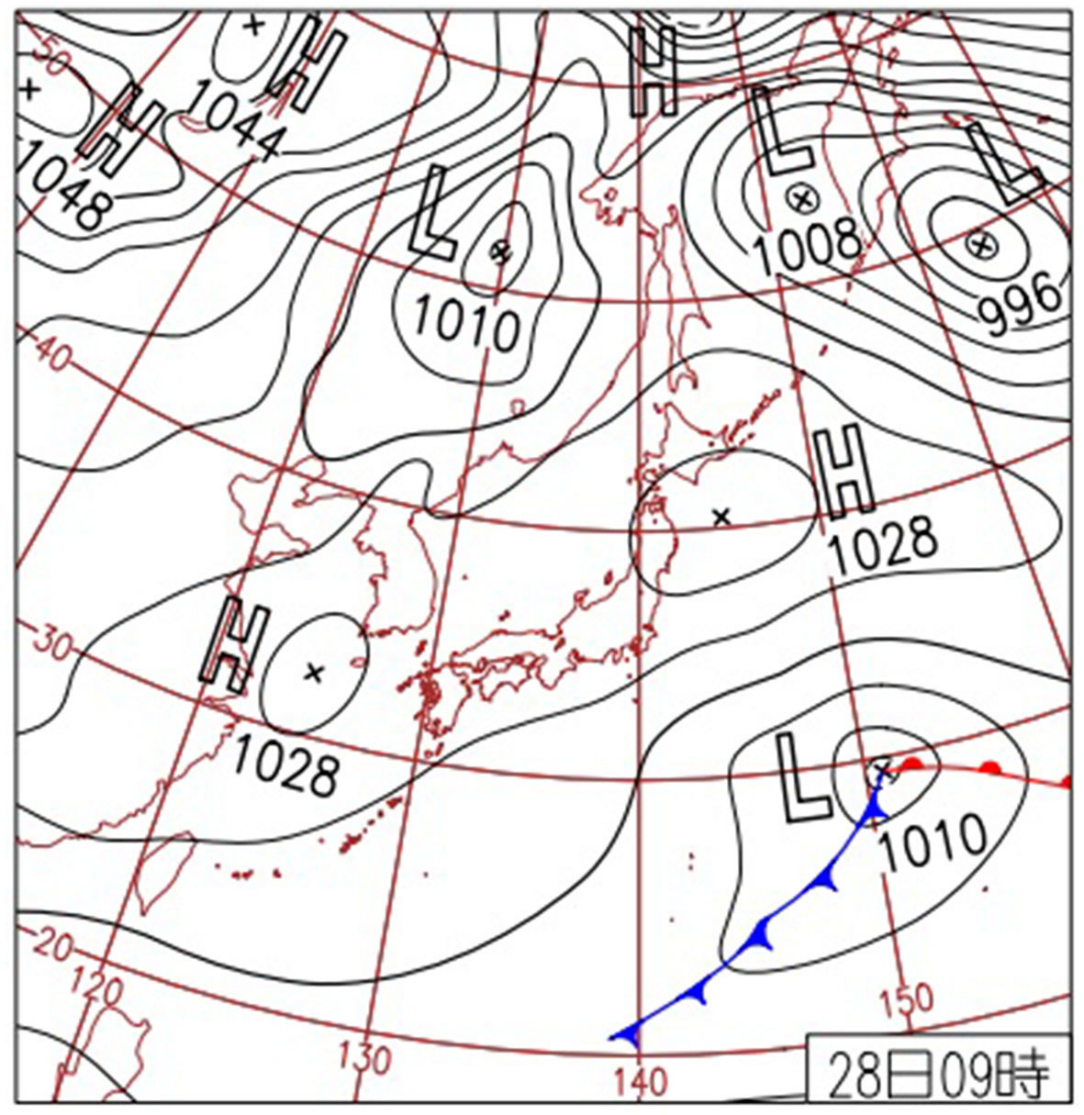
A typical weather chart of the anticyclone belt type around Japan and Asia Pacific. This weather chart is posted on the website of the Japan Meteorological Agency. H, high pressure; L, low pressure.

Following our prior method (1), we investigated the atmospheric pressure distribution on the date closest to the date and time of onset of cerebral infarction in each patient on the Japan Meteorological Agency website^7^ and chose the most similar one from the reported 11 APDPs, which were stationary front (north type) (Fn), stationary front (south type) (Fs), anticyclone belt type (Hb), migratory anticyclone type (Ht), other high-pressure types that cannot be classified as either anticyclone belt type or migratory anticyclone type (H), low-pressure along the south coast type (Ls), pressure trough type (Lt), other low-pressure types (L), southern high-pressure and northern low-pressure type (S), typhoon or extratropical cyclone type (T), and high-pressure to the west and low-pressure to the east type (W). The APDPs were classified based on weather maps around Japan and the Asia-Pacific region. For instance, Figure 1 depicts a typical weather map of the anticyclone belt type. We counted the number of subjects belonging to Group A and Group B for each APDP. Then, for each group, the ratio obtained by dividing the total number as the denominator and the number of subjects for each APDP as the numerator is the appearance rate. Groups A and B were compared for clinical characteristics and the appearance rate of each APDP in each group. The ethics review board of our hospital approved this study (No. 2022-3).

### 2.3. Statistical analyses

Statistical analyses were performed using unpaired t-test and Fisher's exact probability method. For clinical characteristics, we used unpaired t-test to determine which group was statistically significant in each clinical factor. For the appearance rate of each APDP in each group, we used Fisher's exact probability method to determine which group was statistically significant in each APDP. Significance levels were set at a *p*-value of <0.05. Statistics were performed using StatView5.0 software (Abacus Concepts, Berkeley, CA, USA).

## 3. Results

### 3.1. Comparison of Groups A and B for clinical characteristics

Table 1 summarizes the number of subjects, mean age, sex, body mass index (BMI), history of hypertension, history of dyslipidemia, history of diabetes mellitus, history of AF, habitual smoking, habitual alcohol, and ambulance transportation. Mean age, number of subjects with a history of AF, and number of ambulance transportation were statistically significantly higher in Group A than those in Group B (*p* < 0.0001, unpaired *t*-test, respectively). Men/women ratio, BMI, number of subjects with a history of dyslipidemia, number of subjects with a history of diabetes mellitus, and number of subjects with habitual smoking were statistically significantly higher in Group B than in Group A (*p* = 0.0086, *p* < 0.0001, *p* = 0.0062, *p* = 0.0103, *p* = 0.0022, unpaired *t*-test, respectively). No statistically significant difference in the history of hypertension and habitual drinking was observed between Groups A and B.

**Table 1:** Clinical characteristics of patients included in the study.

**Characteristic**	**Embolism (Group A)**	**Thrombosis (Group B)**	***p*-value (unpaired *t*-test)**
Numbers	221	612	not available
Age, y; mean (SD)	78.6 (9.6)	72.6 (12.0)	<0.0001
Men: women	116: 105	383: 229	0.0086
BMI (SD)	22.0 (4.1)	23.3 (4.0)	<0.0001
Hypertension (%)	152 (68.8)	448 (73.2)	0.1972
Hyperlipidemia (%)	29 (13.1)	132 (21.6)	0.0062
Diabetes mellitus (%)	45 (20.4)	179 (29.2)	0.0103
Atrial fibrillation (%)	180 (81.4)	19 (3.1)	<0.0001
Smoking (%)	88 (39.8)	317 (51.8)	0.0022
Drinking (%)	60 (27.1)	204 (33.3)	0.095
Ambulance transportation (%)	175 (79.2)	346 (56.5)	<0.0001

y, year; SD, standard deviation; BMI, body mass index.

### 3.2. Types of atmospheric pressure pattern

**Figure 2:**
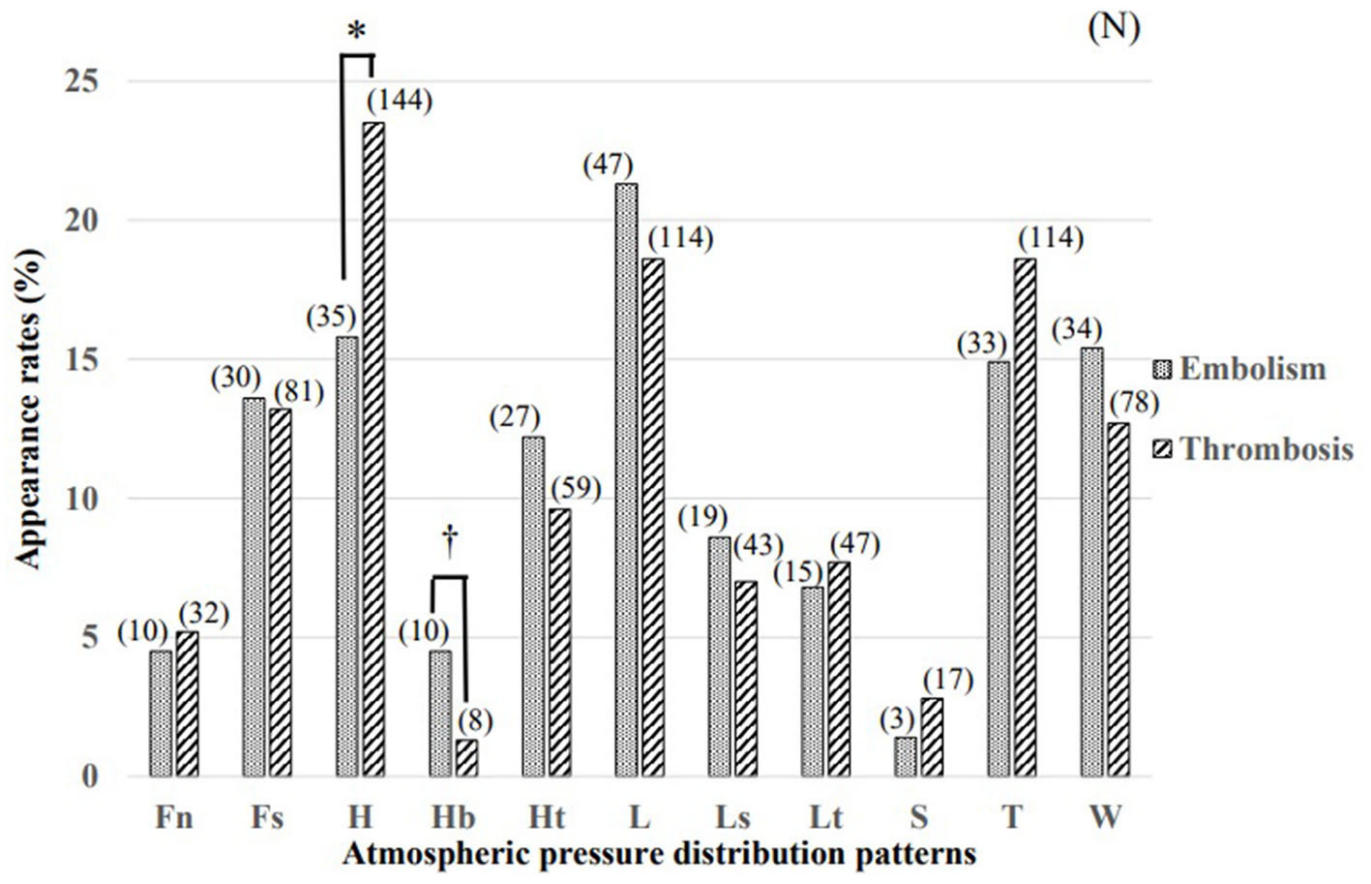
Comparison of atmospheric pressure distribution patterns at the onset of cerebral embolism (Group A) and cerebral thrombosis (Group B). The appearance rate of the other high-pressure type (H) in Group B was statistically significantly higher than that in Group A and the appearance rate of the anticyclone belt type (Hb) in Group A was statistically significantly higher than that in Group B (^*, †^*p* < 0.05, Fisher's exact probability method, respectively). (N), (number of subjects); Fn, stationary front (north type); Fs, stationary front (south type); H, other high-pressure type; Hb, anticyclone belt type; Ht, migratory anticyclone type; L, low-pressure type; Ls, low-pressure along the south coast type; Lt, pressure trough type; S, southern high-pressure and northern low-pressure type; T, typhoon and extratropical cyclone type; W, high-pressure to the west and low-pressure to the east type.

The comparison of the appearance rates in Groups A and B for each APDP is illustrated in Figure 2. Most of the subjects corresponded to only one pattern, but some subjects corresponded to two or three patterns due to overlap of the typhoon or extratropical cyclone type and/or the stationary front (north/south type). In Group A, the appearance rate of the other low-pressure type (L) was highest, followed by that of the other high-pressure type (H), the high-pressure to the west and low-pressure to the east type (W), and the typhoon or extratropical cyclone type (T). In Group B, the appearance rate of the other high-pressure type (H) was highest, followed by that of the other low-pressure type (L), the typhoon or extratropical cyclone type (T), and the stationary front (south type) (Fs). The appearance rate of the other high-pressure type (H) in Group B was statistically significantly higher than that in Group A, and the appearance rate of the anticyclone belt type (Hb) in Group A was statistically significantly higher than that in Group B (*p* < 0.05, Fisher's exact probability method, respectively).

## 4. Discussion

Management of comorbidities such as hypertension, diabetes, dyslipidemia, smoking, drinking, heart disease, and obesity is critical to prevent the development of ischemic stroke. It is known that there are differences in risk factors for each subtype of cerebral infarction. It has been reported that cardiogenic embolism was found to have a higher mean age compared with other categories (6). Hypertension is already known to be the most frequently identified risk factor in all subtypes of ischemic stroke. The Takashima Stroke Registry did not find any significant difference in the prevalence of hypertension across ischemic stroke subtypes (7). Diabetes mellitus has been reported to be more closely associated with small-vessel disease than other subtypes (8). Hypercholesterolemia is a recognized risk factor for stroke due to large-artery atherosclerosis (9). A study examining cerebral infarction by subtypes in Japanese subjects showed that there was a correlation between serum total cholesterol levels and the risk of developing cerebral infarction in large-artery occlusive infarction, but the relationship was weak in other subtypes (10). It is known that BMI may be associated with diabetes mellitus and dyslipidemia, and patients with cardiogenic embolism are often rushed to hospital by ambulance transportation because it is more sudden and serious than cerebral thrombosis. It has been reported that smoking did not show association with any particular subtype (6). In this study, the clinical characteristics of Groups A and B were consistent with these previously reported clinical characteristics of cerebral embolism and cerebral thrombosis except for smoking. The reason for “except for smoking” may be considered to be that there was a large difference in the smoking rate between Reference 6 (7.2%) and our study (48.6%).

Diseases linked to weather changes are collectively called “meteoropathy.” Meteoropathy includes migraines, arthralgia, and asthma, alongside life-threatening myocardial infarction and stroke. In Rome, Italy, climate significantly affects the risk of primary percutaneous coronary intervention for acute myocardial infarction in the current era, with a complex interplay based on the season. Higher risk is expected with lower minimum atmospheric pressure in the preceding days, lower rainfall in winter, greater changes in atmospheric pressure in spring, and higher temperatures in summer (11). Some evidence implies that weather and seasonal variations could affect stroke incidence and outcome. However, in Riyadh, Saudi Arabia, a study observed no impact of weather or seasonal variations on stroke incidence, hospital course, or outcomes (12). Weather conditions often have adverse effects on human health, but this varies by country and region; hence, investigation of the clinical data and weather data in each country and region is necessary.

Japan is an island nation with a temperate climate and has four distinct seasons (spring, summer, autumn, and winter) and characteristic APDPs. An APDP changes the direction of the wind and the flow of the air, also causing the change of the temperature and humidity. In other words, an APDP largely determines wind direction, temperature, humidity, etc., and creates typical weather conditions for each season. There are some characteristic APDPs for each season and the turn of the season, and they are demonstrated on weather charts, which are always reported along with temperature, humidity, wind speed, and wind direction in daily weather forecasts according to data provided by the Japan Meteorological Agency. The Japanese Meteorological Business Law stipulates that forecasts of phenomena must be made by certified and accredited meteorologists, who passed an examination with an average pass rate of 5.5% conducted by the designated examination institution and have been registered by the Director-General of the Japan Meteorological Agency. Then, the first author qualified as a certified and accredited meteorologist of Japan (No. 4282).

Thrombus formation is mediated by the four stages (initiation, amplification, propagation, and stabilization) of the blood coagulation cascade, centered on the key enzyme thrombin generation. AF changes this fine-tuned coagulation system and induces a pro-thrombotic state by the mechanisms outlined in Virchow's triad of blood stasis, hypercoagulability, and endothelial dysfunction, significantly increasing the risk of stroke (13). Dehydration is frequent among stroke subjects and is linked to poor outcomes (14). Ischemic stroke development may be caused by hypoperfusion, renal impairment, and hypercoagulability associated with dehydration status (15–17). Additionally, a recent study indicated that blood urea nitrogen/creatinine > 15 combined with urine specific gravity > 1.010 as a marker of dehydration status was an independent risk factor for long-term poor prognosis of thrombolyzed patients with acute ischemic stroke, implying that rapidly correcting dehydration may be among the therapy strategy in the thrombolyzed patients with acute ischemic stroke (18).

Because of differences in APDPs, weather factors such as temperature, humidity, and wind direction, as well as atmospheric pressure, inevitably change. Hence, the homeostasis of human beings living in such an unstable environment is often upset by dynamic weather changes, and arterial or intra-atrial thrombus may suddenly occur, causing ischemic stroke. In our prior research (1), we categorized the hospitalized severe cases of cerebral infarction that received rt-PA therapy for the first time into embolic and thrombotic infarction and examined the APDP at the onset day. The study demonstrated that the other high-pressure type except for the anticyclone belt type and the migratory anticyclone type was significantly more common in the thrombotic group than in the embolic group, consistent with this study. High-pressure atmospheric conditions, as suggested by the results, might be involved in thrombus formation. The duration of the other high-pressure type is relatively shorter than the migratory anticyclone type and the anticyclone belt type, so short-time pressure fluctuations may be linked to the formation of arterial thrombi.

Some recent papers have reported that prolonged living at higher altitudes, where atmospheric pressure and oxygen concentration decreased, reduced the risk of developing ischemic stroke or dying from it and increased irrigation due to angiogenesis and increased vascular perfusion might be the reason behind improved survival profiles among those living between 2,000 and 3,500 m (19, 20). Based on these reports, it can be also considered that an increase in atmospheric pressure might conversely increase the risk of cerebral infarction, which is very interesting and consistent with the present results.

Alternatively, the anticyclone belt type was significantly more common in Group A than in Group B in this study. This interesting result is the first report in the world. The anticyclone belt type defines a situation where a migratory anticyclone that has passed near Japan intensifies over the east ocean, and two or more high-pressure areas are connected to the east and west to form a belt that covers the vicinity of Japan. It often appears in spring and autumn, and on the Toyama Bay coast in Japan, land–sea breeze circulation is driven and mirages are observed on consecutive days from the coast of Uozu City. Notably, the occurrence of mirages might be associated with atmospheric currents from the southwest and an increase in temperature (21). In the anticyclone belt type, dry and sunny weather lasts for a few days to a week, triggering dehydration and increased blood viscosity in humans. It was also reported that the anticyclone belt type had the second-highest incidence of two or more myocardial infarction patients per day in Hiroshima City (22). This specific weather change in the anticyclone belt type might be a reason why embolic infarction occurred more frequently than thrombotic infarction although there may be differences in the mechanisms of occurrence between cerebral infarction and myocardial infarction. Cerebral embolism often results from dehydration (23) and blood stagnation in the left atrial, specifically in AF patients, and conversely, cerebral thrombosis is caused mainly by arteriosclerosis plus dehydration. Prevention of dehydration is considered to be a significant method for preventing the onset of cerebral infarction, since dehydration is one of the causes of cerebral embolism or cerebral thrombosis.

Even if the APDP is the same, the weather conditions are not always the same among the Hokkaido region, the Kanto region, and the Kyusyu region. Since this is a research study based on clinical data at one facility in Tokyo, Kanto region, the interpretation of this result may not be applicable to all regions in Japan. Similarly, it is unclear whether the results of this survey apply to other countries around the world, as this survey is only about the APDP in Japan and its surroundings. In this study, we first focused on the APDP so as not to see the forest for the trees, but in the future, we will focus on weather factors such as temperature, atmospheric pressure, and humidity, and their changes. Future research is warranted.

## 5. Conclusions

Cerebral embolism and cerebral thrombosis exhibited significant differences in APDPs on the day of onset. In the case of cerebral thrombosis, the other high-pressure type was significantly more frequent, and in the case of cerebral embolism, the anticyclone belt type was significantly more frequent. The onset of cerebral infarction may be affected by differences in APDPs. Since dehydration is among the causes of cerebral embolism or cerebral thrombosis, dehydration particularly in the other high-pressure type or in the anticyclone belt type in Tokyo, Japan, should be prevented. Further research should focus on the other meteorological factors.

## Data availability statement

The raw data supporting the conclusions of this article will be made available by the authors, without undue reservation.

## Ethics statement

The studies involving human participants were reviewed and approved by the Ethics Review Board of Fussa Hospital, Tokyo, Japan. Written informed consent for participation was not required for this study in accordance with the national legislation and the institutional requirements.

## Author contributions

AF: study design, collection and analysis of data, and drafting the manuscript. HK, TF, and AH: revision of the manuscript for important intellectual content. All authors contributed to the article and approved the submitted version.

## Footnotes

^1^ https://www.mhlw.go.jp/stf/english/index.html

^2^Cardioembolism / cardiac pathology (the TOAST classification/ the A-S-C-O/ASCOD phenotypic classification, references 3–5).

^3^Large-artery atherosclerosis / atherothrombosis (the TOAST classification/ the A-S-C-O/ASCOD phenotypic classification, references 3–5).

^4^Small-vessel occlusion / small-vessel disease (the TOAST classification/ the A-S-C-O/ASCOD phenotypic classification, references 3–5).

^5^ https://www.fussahp.jp/index.html

^6^One of medical institutions that accept emergency transport 24 h a day, 365 days a year and provide emergency medical care requiring hospitalization, other than tertiary emergency medical institutions.

^7^ https://www.jma.go.jp/jma/indexe.html
